# Taming Polysulfides in an Li–S Battery With Low-Temperature One-step Chemical Synthesis of Titanium Carbide Nanoparticles From Waste PTFE

**DOI:** 10.3389/fchem.2021.638557

**Published:** 2021-03-11

**Authors:** Suyao Liu, Jun Luo, Yuting Xiong, Zhe Chen, Kailong Zhang, Guofeng Rui, Liangbiao Wang, Guang Hu, Jinlong Jiang, Tao Mei

**Affiliations:** ^1^ Key Laboratory for Palygorskite Science and Applied Technology of Jiangsu, National & Local Joint Engineering Research Center for Mineral Salt Deep Utilization, School of Chemical Engineering, Huaiyin Institute of Technology, Huaian, China; ^2^ Hubei Collaborative Innovation Center for Advanced Organic Chemical Materials, Ministry-of-Education Key Laboratory for the Green Preparation and Application of Functional Materials, Hubei Key Laboratory of Polymer Materials, School of Materials Science and Engineering, Hubei University, Wuhan, China; ^3^ School of Chemistry and Environment Engineering, Jiangsu University of Technology, Changzhou, China

**Keywords:** chemical synthesis, titanium carbide, X-ray diffraction pattern, nanoparticles, Li–S batteries

## Abstract

In this work, titanium carbide (TiC) nanoparticles have been successfully synthesized at much lower temperatures of 500°C using cheaper starting materials, such as waste polytetrafluoroethylene (PTFE) (carbon source) and titanium and metallic sodium, than the traditional carbothermal reduction of TiO_2_ at 1,800°C. An XRD pattern proved the formation of face-centered cubic TiC, and TEM images showed the obtained TiC nanoparticles with an average size of approximately 50 nm. In addition, the separator coated with TiC nanoparticles as an active material of interlayer effectively mitigates the shuttling problem by taming the polysulfides in Li–S batteries compared with a traditional celgard separator. The assembled cell realizes good cycling stability with 501 mAh g^−1^ and a low capacity fading of 0.1% per cycle after 300 cycles at 1 C due to high utilization of the sulfur-based active species.

## Introduction

Transition metal carbides have recently received much attention for their important applications in the mechanical and aerospace industries (Das et al., 2002; Rasaki et al., 2018). Among transition metal carbides, titanium carbide (TiC) is an important non-oxide ceramic material due to its good chemical stability, extreme hardness, high melting point (about 3,200°C), high Young’s modulus, low density (4.93 g/ml), and good electrical and thermal conductivity (Wang et al., 2012a; Wang et al., 2012b; Xiao et al., 2016). Recently, TiC has been considered for use in energy storage due to its outstanding characteristics (Liu et al., 2018; Cui et al., 2019; Cai et al., 2020; Geng et al., 2021). Specifically, in an Li–S battery, the synthesized TiC can be used to enhance the cycling performance through the strong polar binding interactions with sulfur species and can better suppress the diffusion of lithium polysulfides (LiPS) compared with other materials (Zhang et al., 2016; Cui et al., 2018; Zhang et al., 2018; Zhang et al., 2019). The strong chemical sorption and high electrical conductivity make it an ideal sulfur host for Li–S batteries. Mei and co-workers successfully prepared a TiC–TiO_2_/S composite and the result shows that the cathode with a TiC–TiO_2_/S composite delivers a high initial discharge specific capacity of 1,218 mAh g^−1^ at 1 C rate (Cui et al., 2019). In the TiC–TiO_2_/S composite, TiC can not only chemically bond with polysulfides but also improve the electrical conductivity of the assembled cells (Cui et al., 2019; Geng et al., 2021). The result also shows that it can promote the conversion kinetics of LiPS to final Li_2_S_2_/Li_2_S during the electrochemistry of Li–S batteries (Zhang et al., 2020). Based on this, Zhang designed and synthesized multi-yolk/shell structured TiC@C nanofibers as a strong sulfur host. Owing to its chemisorption, active electrocatalysis, and high electrical conductivity properties, the as-prepared sulfur-based cathode shows an unforgettable areal capacity of 6.81 mA h cm^−2^ over 800 cycles with a high sulfur loading (10.5 mg). Therefore, TiC nanoparticles are a popular candidate for inhibiting the LiPS shutting effect (the main problem in Li–S batteries) (Cai et al., 2020).

Traditionally, the industrial method of producing TiC is the carbothermal reduction of TiO_2_ at high temperatures (Berger et al., 1999; Cai et al., 2020). Over the past decades, various other methods have been reported to synthesize TiC, including self-propagating high-temperatures synthesis (SHS) (Song et al., 2009), mechanochemical synthesis (Lyakhov et al., 2018), sonochemical (Sivasankaran and Kumar, 2015), melting‒casting (Liu et al., 2014), improved carbothermal reduction (Yu et al., 2020), metallic thermal reduction (Lee et al., 2004), spark plasma sintering technique (Saba et al., 2018), chemical vapor deposition (Qi et al., 2000; Wokulski and Wolaska, 1983), mechanical alloying (MA) (Razavi et al., 2007), and so on (Wang et al., 2016; Fei et al., 2020). As everyone knows, waste plastic is solid waste with a high carbon content. Therefore, the waste plastic can be used as raw materials to synthesize high-valued carbon-based materials and carbides (Wang et al., 2017; Wang et al., 2018; Dai et al., 2019; Wang et al., 2019a; Wang et al., 2019b; Wang et al., 2019c; Wang et al., 2019d). In this study, we have developed a new method to synthesize TiC nanoparticles by using waste PTFE as a carbon source at a low temperature of 500°C. The formation mechanism of TiC has been discussed preliminarily. And its taming of LiPS by coating TiC nanoparticles on one side of the separator in an Li–S battery has been investigated. The result proves that the assembled cell achieved a higher specific capacity at the same rate and lower capacity loss in long-cycle performances compared with a traditional celgard separator.

## Experiment

### Synthesis of TiC Nanoparticles

The titanium powder, metallic sodium, sulfur powder, N-methyl-2-pyrrolidone (NMP), and Li_2_S powder used in experiments were purchased from Sinopharm Chemical Reagent Co., Ltd. Super P and polyvinylidene fluoride (PVDF, HSV900) were bought from Lizhiyuan Store (Taiyuan city, Shanxi Province, China). All chemicals were used directly without further treatment. Metallic titanium (0.40 g), waste PTFE (obtained from waste teflon‒lined, 0.50 g), and excess metallic sodium (1.00 g) were put into a stainless autoclave of 22 ml capacity. After sealing, the stainless autoclave was kept at 500°C for 10 h and then cooled to 30°C. The product was collected from the autoclave and washed with absolute ethanol and diluted with HCl several times to remove the byproducts and unreacted sodium. The obtained black powder was dried at 70°C for several hours under vacuum.

### Preparation of Sulfur Electrode

Melt-diffusion method was conducted to prepare the Super P/S (C/S) composite. A mixture of Super P and S (with a weight ratio of 4:6) was grounded in an agate mortar for 20 min. Then, the collected powder was transferred into a quartz tube and heated at 155°C for another 24 h to obtain a C/S composite. Subsequently, the C/S composite and PVDF were mixed (with a weight ratio of 9:1) to form homogeneous slurry with drops of NMP. The slurry was coated onto one side of the aluminum foil, and the coated aluminum foil was dried in a vacuum oven at 70°C for 12 h. Lastly, the dried coated aluminum foil was punched into discs (with a diameter of 12 mm). The sulfur loading is about 1.6–2.0 mg cm^−2^.

### Preparation of Modified Separator

The obtained TiC (80 mg), Super P (10 mg), and PVDF (10 mg) binder were grinded for 10 min before being dispersed in NMP. After adding NMP, further sequential grinding for 10–15 min was taken to form homogeneous slurry. Then the slurry was coated on one side of a (celgard 2500) separator. The modified separator was dried at 40°C under vacuum for 10 h. Finally, the modified separator was cut into a circle with a diameter of 19 mm.

### Adsorption Study of TiC

Li_2_S_6_ solution was prepared by mixing sulfur powder and Li_2_S with a molar ratio of 5:1 in tetrahydrofuran (THF) solvent, followed by stirring at 40°C for 24 h in a confined glass bottle in an Ar-filled glove box. Twenty milligram of TiC powder was dispersed in 10 ml Li_2_S_6_/THF solution with a concentration of 5 mmol L^−1^. The mixtures were kept standing for 2 h to observe the color change.

### Characterization

The final product (TiC) was characterized by X-ray powder diffraction (XRD, Philips X’Pert diffractometer with Cu-Kα radiation *λ* = 1.54178 Å) and field-emission scanning electron microscopy (JEOL-JSM-6700F). High Resolution-Transmission electron microscopy (HR-TEM) images and selected area electron diffraction (SAED) of TiC nanoparticles were performed on a JEOL-2010 transmission electron microscope. The photos of LiPS adsorption experiment and separator coated with TiC nanoparticles were taken by iPhone 7.

### Electrochemical Measurements

Electrochemical measurements were carried out with coin-type 2032 half cells in an Ar-filled glove box (H_2_O < 0.1 ppm, O_2_ < 0.1 ppm). The Li–S cell was assembled with C/S cathode, one piece of separator, and lithium metal with 1 mol L^−1^ bis (trifluoromethane) sulfonimide lithium salt (LiTFSI) dissolved in a mixture of 1,2-dimethoxyethane (DME) and 1,3-dioxolane (DOL) (v/v = 1/1) containing LiNO_3_ (2 wt%). The cells were cycled in the voltage range of 1.7–2.8 V. The electrochemistry impedance spectroscopy (EIS) was tested on a CHI660e electrochemical workstation (100 kHz–0.01 Hz) using an open circuit voltage. The cycling performance and rate performance were based on the galvanostatic test (1 C = 1,675 mA g^−1^).

## Results and Discussion

**FIGURE1:**
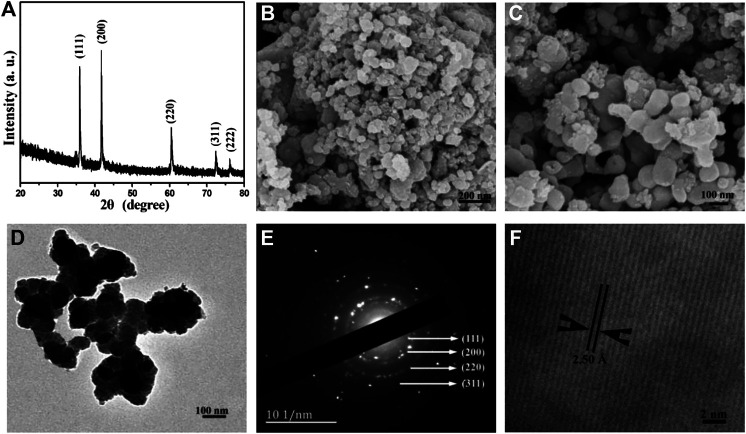
**(A)** XRD pattern, **(B)** Low-magnification SEM image, **(C)** High-magnification SEM image, **(D)** TEM image, **(E)** SAED pattern, and **(F)** HR-TEM image of the as-obtained TiC nanoparticles.

The XRD pattern of the as-obtained product TiC (shown in Figure 1A) was used to investigate the crystal structures. All the diffraction peaks with strong diffraction intensity centered at 2*θ* = 35.91°, 41.73°, 60.45°, 72.37°, and 76.16°, which can be indexed as the (111), (200), (220), (311), and (222) diffraction planes of the face-centered cubic titanium carbide, respectively. The refined lattice parameter *a* = 4.3253 Å is extracted from the XRD pattern, which is close to the literature value of *a* = 4.3270 Å (JCPDS card No. 65-0242). No other diffraction peaks in the XRD pattern from impurity have been found. The XRD pattern proves the well-crystallized face-centered cubic structure of TiC powder. TiC has been obtained from the reaction of metallic titanium, waste PTFE, and metallic sodium under the present synthetic conditions.

SEM is used to investigate the morphology and size of the product. A representative SEM image of the obtained TiC product is shown in Figure 1B, which apparently shows that the product is mainly composed of nanoparticles with a diameter of 30–100 nm. From a high magnification SEM image (Figure 1C), it can be clearly observed that TiC nanoparticles aggregate into chainlike nanostructures.

A typical TEM image of the TiC sample is shown in Figure 1D. The size of the obtained TiC nanoparticles shows an average of ∼50 nm. Furthermore, the highly crystalline nature of the product was confirmed by the SAED pattern shown in Figure 1E. The diffraction rings can be indexed as (111), (200), (220), (311), and (222) reflections according to the cubic structure of polycrystalline TiC, which is consistent with the XRD result. HR-TEM is used to further investigate the detailed structure of nanocrystalline TiC. As shown in Figure 1F, the lattice spacing is measured to be 2.50 Å, which corresponds to the (111) plane in cubic phase TiC.

In our experiment, the approach to synthesize TiC nanoparticles is based on a chemical reaction with metallic sodium, waste PTFE, and metallic titanium. The chemical reaction can be expressed as follows:

$$2\text{Na}+\text{Ti}+1/\text{n}{\left[{\text{CF}}_{2}\right]}_{\text{n}}\to 2\text{NaF}+\text{TiC}$$ (1)

During the reaction process, waste PTFE broke down by metallic sodium to produce carbon with the increasing reaction temperature (Eq. 2). Then, TiC nanoparticles can be prepared from the following chemical reaction with carbon atoms and metallic titanium due to the high reactivity of the newly formed carbon atoms (Eq. 3). The chemical reactions in the autoclave may be expressed as follows:

$$2\text{Na}+1/\text{n}{\left[{\text{CF}}_{2}\right]}_{\text{n}}\to 2\text{NaF}+\text{C}$$ (2)

Ti+C→TiC (3)

As the standard molar enthalpies formation of NaF is negative (Δ_r_ *H*_m_,_NaF_ = −572.63 kJ mol^−1^), a very large amount of heat has been generated in the reaction Eq. 2, promoting the formation of TiC nanoparticles at a low reaction temperature of 500°C.

**FIGURE 2:**
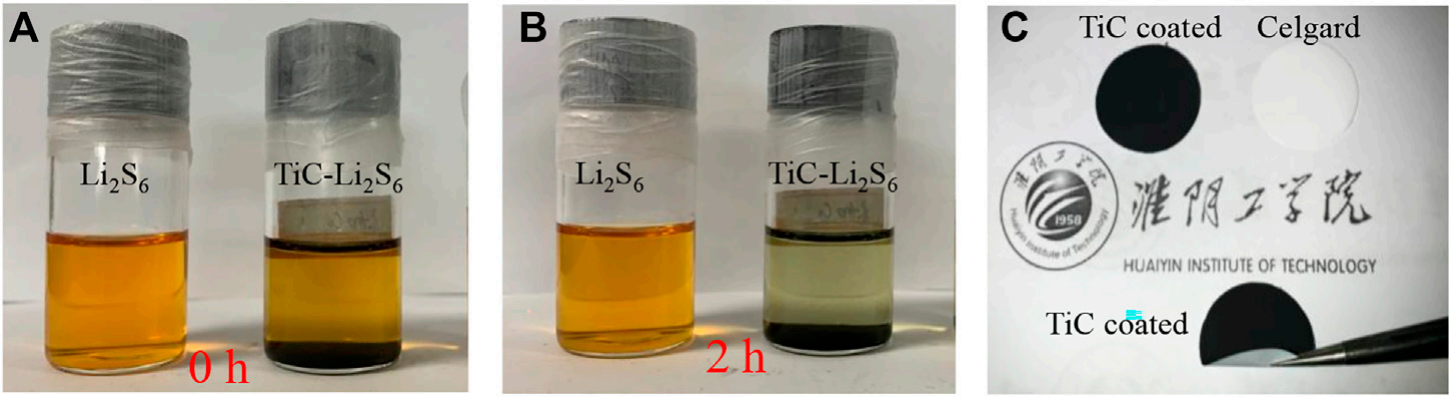
The optical images of the LiPS adsorption experiment: **(A)** Before and **(B)** After rest for 2 h, **(C)** Photographs of the of the separator coated with TiC nanoparticles and bend test.

To further study the interactions between TiC and LiPS, the visual effect is given by dispersed TiC nanoparticles in 5 mmol L^−1^ LiPS solution. As shown in Figures 2A,B, the color of the bottle labeled with TiC-Li_2_S_6_ changes from dark yellow to almost transparent after adsorption for 2 h, while the bottle labeled with Li_2_S_6_ remains unchanged. This result indicates that as-prepared TiC nanoparticles have great potential in inhibiting LiPS shuttling in Li–S batteries (Zhang et al., 2020). Considering the merit of the coating separator, TiC nanoparticles were coated on one side of a celgard 2500 separator with Super P and PVDF serving as a functional separator for coin cells. The photos of both the separator coated with TiC nanoparticles and the common celgard separator are shown in Figure 2C. The reversible bend test also gives strong proof of an excellent adhesion of TiC to the separator without any powder. Therefore, the dissolved LiPS cannot easily shuttle through the separator (Xu et al., 2019). This also provides a great potential in keeping the intact structure for a long performance period.

**FIGURE 3:**
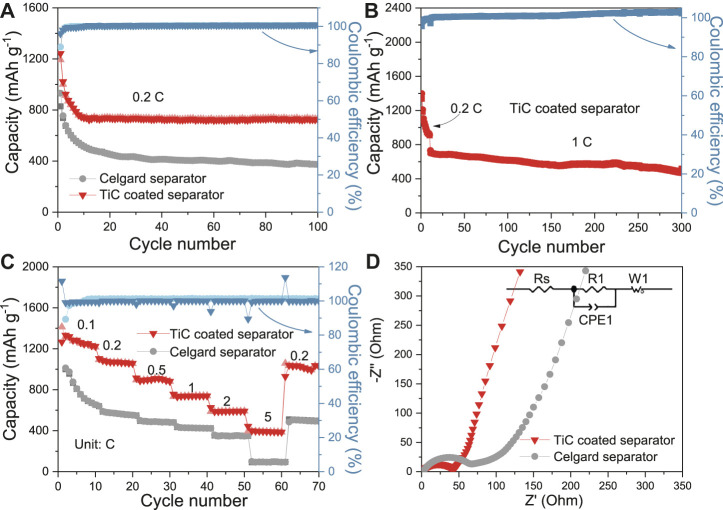
**(A)** Cycling performance at 0.2 C, **(B)** Long term cycling performance at 1 C based on C/S cathode and TiC coated separator, **(C)** Multi-rate performance of the cells based on C/S cathode with celgard and TiC-coated separators, **(D)** EIS of the cells based on C/S cathode with celgard and TiC-coated separators before cycle.

Using TiC nanoparticles to construct a separator modifier, cycling performances and rate performances were systemically studied. As exhibited in Figure 3A, the battery using the same cathode but with a TiC-nanoparticles-coated-separator delivers a high initial specific capacity of 1,242 mAh g^−1^, and the specific capacity can remain at 736 mAh g^−1^ after 100 cycles at 0.2 C rate, which are higher than those of the battery composed of a C/S cathode with a celgard separator (827 and 373 mAh g^−1^). Moreover, the long-term cycling performance was also researched (Figure 3B). The battery-based TiC coated separator shows specific capacity of 768 mAh g^−1^ at 1 C after the first ten cycles of activation, and maintains a specific capacity of 501 mAh g^−1^ with a low capacity fading of 0.1% per cycle after 300 cycles. In addition, multi-rate performances were also shown in Figure 3C. Specifically, when the current rate increases from 0.1 to 0.2, 0.5, 1, 2, and 5 C, the discharge specific capacities of the cell with a TiC-nanoparticles-coated-separator can be higher and more stable at around 1,412, 1,118, 914, 756, 652, and 399 mAh g^−1^, respectively. Even at a high current density of 5 C, the discharge capacity can also still maintain at about 400 mAh g^−1^. As the current density returned to 0.2 C, the reversible capacity can return to 1,035 mAh g^−1^, suggesting a good reversibility. Moreover, the EIS electrochemical performance of the battery based on cells with a TiC-nanoparticles-coated-separator and common celgard separator before cycle was investigated and the result is shown in Figure 3D. In general, the low frequency slope and high frequency semicircle can be attributed to Warburg impedance (slope) and charge transfer resistance (semicircle), respectively (Zhang et al., 2020). Therefore, as shown in Figure 3D, it is obvious that the Warburg coefficient (slope) is very close in the low frequency region. However, the cell assembled with a TiC-nanoparticles-coated-separator illustrates a more stable and lower charge transfer resistance (46.2 Ω) than the celgard separator (70.6 Ω) due to the good conductivity and stable structure of a TiC-nanoparticles-coated-separator.

Benefiting from the unique characteristics of TiC nanoparticles, the modified separator greatly improved lithium storage performances for Li–S batteries. On one hand, nano-sized TiC can effectively contact aqueous-like LiPS and facilitate good interface interaction with them. On the other hand, TiC materials as interlayers can capture the shuttled LiPS and catalyze them tightly, contacting with cathode. Thus, the enhanced electrochemical reactivity and electronic conductivity of TiC nanoparticles result in less capacity fading during rate performance and a longer working time.

## Conclusion

TiC nanoparticles have been successfully synthesized using a lower temperature of 500°C, cheaper starting materials, and waste PTFE. This method is a more environmentally friendly way to recycle waste PTFE and requires less energy compared with the traditional carbothermal reduction of TiO_2_ at 1,800°C. As a taming material for LiPS in Li–S batteries, cells with a TiC-nanoparticles-coated-separator demonstrate enhanced electrochemical properties due to their high conductivity and polar property of TiC.

## Data Availability

The original contributions presented in the study are included in the article/Supplementary Material, further inquiries can be directed to the corresponding authors.
